# Complete Genome Sequence of Stenotrophomonas maltophilia 1800, a New Bacterial Strain with Potential for Bioremediation of Oil-Contaminated Environments

**DOI:** 10.1128/mra.01116-21

**Published:** 2022-02-17

**Authors:** Annela Semai, Frédéric Plewniak, Joanna Lledo, Gwenolah Annonay, Céline Vandecasteele, Céline Lopez-Roques, Philippe N. Bertin

**Affiliations:** a Génétique Moléculaire, Génomique et Microbiologie, Université de Strasbourg, Strasbourg, France; b INRAE, GeT-PlaGe, Genotoul, Castanet-Tolosan, France; University of Southern California

## Abstract

Stenotrophomonas maltophilia strain 1800 was isolated from the effluent of an industrial oil refinery in Algeria. Its genome was sequenced using Illumina MiSeq (2 × 150-bp read pairs) and Oxford Nanopore (long reads) technologies and assembled using Unicycler. It is composed of one chromosome of 4.83 Mb.

## ANNOUNCEMENT

Most often described as an opportunistic pathogen (1), Stenotrophomonas maltophilia has also been shown to produce various biosurfactants/bioemulsifiers and to degrade diesel or motor oil (2, 3).

Stenotrophomonas maltophilia strain 1800 was isolated using the suspension-dilution method from a petrochemical refinery near Oran, Algeria (35°49′49.6″N, 0°19′31.6″W), on mineral salt medium (MSM) plates supplemented with trace elements and crude oil (1% vol/vol) (4). DNA was extracted from an LB liquid medium culture incubated for 72 h at 30°C using the MasterPure complete DNA and RNA purification kit (Epicentre) and used for both sequencing libraries.

DNA sequencing (DNA-seq) libraries were prepared following Illumina’s protocols for the Illumina TruSeq Nano DNA low-throughput (LT) library prep kit. The library quality was assessed using an advanced analytical fragment analyzer (Agilent), and the libraries were quantified by quantitative PCR (qPCR) using the Kapa library quantification kit (Roche). Paired-end sequencing (2 × 150 bp) was performed on an Illumina MiSeq instrument with the Illumina MiSeq Reagent Kit v2 Micro.

Oxford Nanopore Technologies (ONT) libraries were prepared according to the manufacturer’s instructions for 1D native barcoding genomic DNA (EXP-NBD104 and SQK-LSK109 kits). The DNA was quantified using the Qubit double-stranded DNA (dsDNA) high-sensitivity (HS) assay kit (Life Technologies), and the purity was determined using a NanoDrop instrument (Thermo Fisher Scientific). The size distribution was assessed using the fragment analyzer (AATI) high-sensitivity DNA fragment analysis kit. The DNA was purified using AMPure XP beads (Beckman Coulter) and sheared at 20 kb (speed 32) using the Megaruptor III system (Diagenode). Following DNA damage repair, end repair, a dA-tailing step, and sample-specific index ligation, the library was loaded onto an R9.4.1 revD flow cell and sequenced on a GridION instrument at 0.013 pmol within 48 h using MinKNOW v21.05.12 and Guppy v5.0.12 for base calling.

Default parameters were used for all software unless otherwise specified. Short reads with an average quality lower than 35 were filtered out using BBDuk v38.91 from JGI’s BBTools suite (https://jgi.doe.gov/data-and-tools/bbtools/bb-tools-user-guide). Long reads with an average quality greater than 9 were further filtered using Filtlong v0.2.1 (https://github.com/rrwick/Filtlong), using the short reads as an external reference for quality assessment. The best reads longer than 20 kb were kept for a cumulative length of 500 Mb.

Hybrid assembly with Unicycler v0.4.8 (5) of the long and short filtered reads yielded one complete circular 4,837,110 bp-long contig with a GC content of 66.19%. The average coverage estimated using QUAST v5.0.2 (6) was 136×, with 100% of reads mapping back onto the assembly. All 659 marker genes for *Xanthomonadaceae* were found using CheckM v1.1.3 (7) in the assembly, suggesting that the genome was 100% complete. Potential contamination was insignificant (0.11%), and no strain heterogeneity was identified. A GToTree v1.5.47 phylogenomic analysis using 172 gammaproteobacterium single-copy genes in 204 S. maltophilia reference strains and Stenotrophomonas pictorum JCM 9942 as an outgroup suggested that S. maltophilia 1800 belongs to the S. maltophilia complex genogroup F (8, 9).

MicroScope automatic genome annotation (10) predicted 4,671 coding sequences (CDS), 70 tRNAs, and 13 rRNA genes. BLASTP (11) searches using Geobacillus thermoleovorans B23 LadAα, LadAβ, and LadB as queries identified one putative LadAβ alkane monooxygenase in S. maltophilia 1800 (12).

### Data availability.

The raw reads and assembly of the Stenotrophomonas maltophilia 1800 genome have been deposited in DDBJ/EMBL/GenBank under BioProject accession number PRJEB48474. The raw reads are available in the SRA under accession numbers ERR7198268 for the MiSeq paired-end reads and ERR7198269 for the ONT long reads. The sequence of the chromosome is available in RefSeq under the accession number NZ_OU943334.
